# Mechanical Performance of a Ballastless Track System for the Railway Bridges of High-Speed Lines: Experimental and Numerical Study under Thermal Loading

**DOI:** 10.3390/ma14112876

**Published:** 2021-05-27

**Authors:** Yingying Zhang, Lingyu Zhou, Akim D. Mahunon, Guangchao Zhang, Xiusheng Peng, Lei Zhao, Yahui Yuan

**Affiliations:** 1 Department of Civil Engineering, Central South University, 68 South Shaoshan Road, Changsha 410075, China; z184812279@csu.edu.cn (Y.Z.); zhoulingyu@csu.edu.cn (L.Z.); mahunonakim@csu.edu.cn (A.D.M.); zhang24@csu.edu.cn (G.Z.); pengxiusheng@csu.edu.cn (X.P.); 194811021@csu.edu.cn (Y.Y.); 2 Central-South Architectural Design Institute Co., Ltd, 2 Central South 2nd Road, Wuhan 430071, China; 3 National Engineering Laboratory for High Speed Railway Construction, Central South University, Changsha 410075, China

**Keywords:** railway bridges, high-speed railway, ballastless track structures, thermal load, relative displacements, stress analysis, testing and modeling

## Abstract

The mechanical performance of China Railway Track System type II (CRTS II) ballastless track suitable for High-Speed Railway (HSR) bridges is investigated in this project by testing a one-quarter-scaled three-span specimen under thermal loading. Stress analysis was performed both experimentally and numerically, via finite-element modeling in the latter case. The results showed that strains in the track slab, in the cement-emulsified asphalt (CA) mortar and in the track bed, increased nonlinearly with the temperature increase. In the longitudinal direction, the zero-displacement section between the track slab and the track bed was close to the 1/8L section of the beam, while the zero-displacement section between the track slab and the box girder bridge was close to the 3/8L section. The maximum values of the relative vertical displacement between the track bed and the bridge structure occurred in the section at three-quarters of the span. Numerical analysis showed that the lower the temperature, the larger the tensile stresses occurring in the different layers of the track structure, whereas the higher the temperature, the higher the relative displacement between the track system and the box girder bridge. Consequently, quantifying the stresses in the various components of the track structure resulting from sudden temperature drops and evaluating the relative displacements between the rails and the track bed resulting from high-temperature are helpful in the design of ballastless track structures for high-speed railway lines.

## 1. Introduction

By the end of 2020, the total mileage of the Chinese HSR exceeded 35,000 km [1]. With this rapid development of HSR, damage problems and structural drawbacks [2,3,4,5,6,7,8] have been observed in many high-speed lines. During the service period, HSR ballastless slab tracks are not only affected by traffic load or foundation deformation, but also by environmental thermal conditions. The existing literature shows that the influence of temperature on HSR slab tracks cannot be ignored [9,10,11,12,13]. Thermal action includes the overall temperature action and the temperature–gradient action. The overall temperature action causes the expansion, while temperature gradient causes flexural deformations in the structure [14,15,16,17,18,19,20]. The changes in ambient temperature cause temperature changes within the track structure, and a complex thermal field is generated inside the structural system [21,22,23,24,25]. Regarding the thermal field in the track slab, Gao Liang et al. [19] studied the characteristics of track slab surface temperature following the change of ambient temperature through long-term temperature monitoring and formulated a relationship between track slab surface temperature and ambient temperature using a quartic polynomial and exponential distribution model. Dai Gonglian et al. [20] proposed a temperature distribution model and a transverse and vertical thermal gradient mode of ballastless track using long-term temperature data and statistical analysis. Ou Zumin et al. [21] formulated the extreme value probability distribution model of track slab temperature based on probability demand and theoretical derivation; they concluded that a reasonable value of thermal load could be determined using the model. Zhao Lei et al. [22] analyzed the distribution of two-dimensional temperature fields in CRTS Ⅱ ballastless tracks and put forward the three-dimensional distribution pattern of horizontal and vertical temperature in track systems by carrying out the temperature test under typical high-temperature weather in a laboratory. Based on the experimental study, Zhang Guangchao et al. [23] observed that the stiffness of the track structure gradually degenerated under cyclic thermal load, which induced the separation between the track slab and the CA mortar layer. On the other hand, in view of the damage mechanism of ballastless track structure due to thermal load, Chen Long et al. [26] classified interlayer damage of the track structure based on the bond slip model and showed that, under thermal gradient action, interlayer damage mainly occurs at the edge of track slab, and the increase in interlayer interface bond strength can reduce the interlayer damage. Yang Linqi et al. [27] introduced material static and fatigue damage constitutive models, and simulated interlayer interface damage degradation using an interface cohesion model; they formulated a three-dimensional nonlinear finite element model, and discussed the time-dependent behavior of the ballastless track–bridge structural system under traffic load, which was instrumental in studying the ballastless track structure damage under thermal load.

The existing literature [19,20,21,22,23,24,25,26,27] mainly discusses ballastless track temperature field prediction and the damage characteristics of the track structure under thermal load. There are, however, scant studies on the internal causes of ballastless track damage under thermal load. Deformation characteristics of CRTS Ⅱ ballastless tracks on an HSR bridge under thermal load are still lacking. Therefore, in this project, a one-quarter- scaled specimen of a CRTS II slab ballastless track suitable for HSR bridges was constructed and a thermal field was re-created in the lab. At the same time, interlayer displacements and strains of the structural system were analyzed, and the mechanical performance of the ballastless slab track structure under thermal load was studied.

## 2. Test Overview

According to the similarity principle [28,29], a one-quarter-scaled specimen of a ballastless track slab was designed and built. The concrete, emulsified asphalt mortar [30,31,32,33,34,35], steel bar, and other materials used in the specimen are consistent with that of the prototype structure.

**Figure 1:**
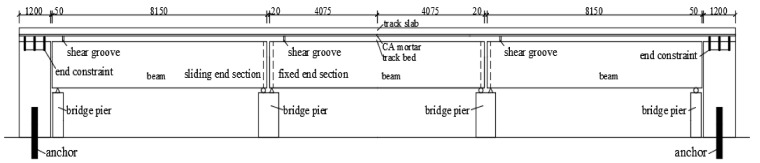
Elevation of the beam specimen (unit: mm).

**Figure 2:**
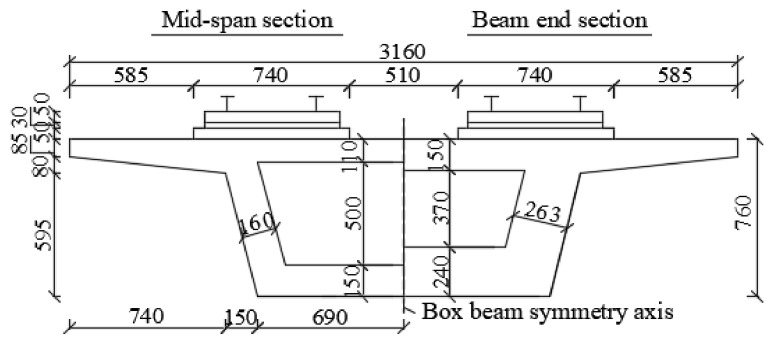
Cross section of the beam specimen (unit: mm).

A drawing with the side view of the structure is provided in Figure 1, with (top to bottom) rails, fasteners, track slab, mortar layer, track bed, bridge beams and bridge piers. Each of the three spans is 8.15 m long. To take care of the continuity in the longitudinal direction, solid RC blocks (each 1.2 m long and with a 20 ton mass) were cast at the extremities of the single span under examination. Bolts were used to anchor the blocks to the foundation. In turn, the track bed was anchored to the blocks via steel bars. The geometry of the cross section is shown in Figure 2.

### 2.1. Similarity Theory

As from Table 1 and Table 2 and Equations (1)–(4), the test appears to be a reference for the actual structure subjected to thermal loads. In other words, for the same thermal load, the strains measured in the test and the stresses derived from the numerical analysis are the same as those in the actual structure, and the displacements measured on the scaled-down specimen are one-quarter of the actual displacements.

(1) $$\nabla T=\frac{\Delta T}{L}({}^{\circ}C/m)$$

(2) $$\varepsilon_{x}=\frac{1}{E}[\sigma_{x}-\mu \;(\sigma_{y}+\sigma_{z})]+\alpha \Delta T$$

(3) $$\sigma_{x}=-\frac{E(\alpha \Delta T)}{1-2\mu }$$

(4) $$c_{\sigma_{x}}=\frac{\sigma_{x}}{\sigma_{x}^{ꞌ}}=1;c_{\varepsilon_{x}}=\frac{\varepsilon_{x}}{\varepsilon_{x}^{ꞌ}}=1$$

$\varepsilon_{x}$: strain in the *x*-direction, με

*E*: elastic modulus, GPa

μ: Poisson’s ratio

α: linear expansion coefficiet,${}^{\circ}C^{-1}$

ΔT: thermal difference in the vertical direction,°C

∇*T*: thermal gradient, °C/m.

### 2.2. Temperature Sensors

**Figure 3:**
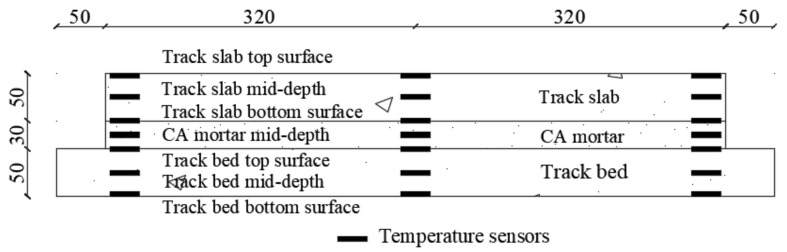
Distribution of the temperature sensors and strain gauges (unit: mm).

JMT-36B temperature sensors with an accuracy of ±0.1 °C were used for collecting temperature data. Temperature monitoring points were arranged at beam end sections and mid-span sections; three rows of temperature monitoring points are displayed in the transverse direction, and three layers of temperature measuring points are arranged at the upper, middle and lower levels of each track layer in the vertical direction. The distribution of temperature sensors and strain gauges is shown in Figure 3. During the heating process, the thermal field was monitored at 3 min intervals, while during the cooling process, 5 min intervals were adopted.

### 2.3. Arrangement of Strain Sensors

**Figure 4:**
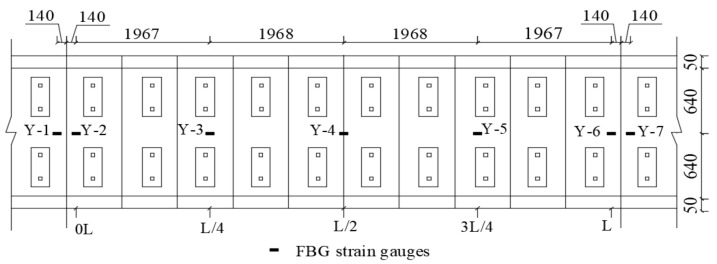
Layout of FBG strain gauges in the track layers (unit: mm).

Fiber Bragg grating (FBG) strain gauges were used to collect strain data, and the precision of the strain sensors was 1 με. The FBG strain gauges were embedded before concrete and CA mortar were cast. Figure 4 shows the layout of FBG strain gauges in the longitudinal direction of the beam, where strain gauges are arranged on the central axis of both track lines in the mid-span section, L/4 sections, ends support section, and 140 mm away from both support sections. The points where the strains were monitored in the transverse direction were arranged at mid-depth of each layer of the track.

### 2.4. Arrangement of Displacement Monitoring Points

To measure the interlayer displacement of track structure and the overall displacement of the support beam, 5G10X series linear displacement gauges with an accuracy of 5/1000 were used. The displacement gauges were arranged at the support sections, L/4, 3L/4, and mid-span sections of the test beam specimen. Linear variable differential transformer (LVDT) displacement gauges were arranged at the aforementioned sections of each track line to measure the relative longitudinal and relative vertical displacement between different track layers and between the track system and support beam.

**Figure 5:**
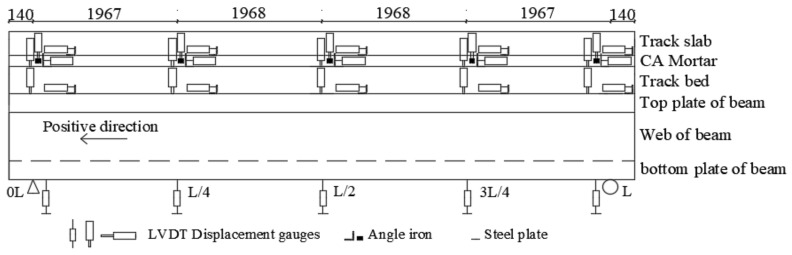
Layout of LVDT displacement gauges (unit: mm).

**Figure 6:**
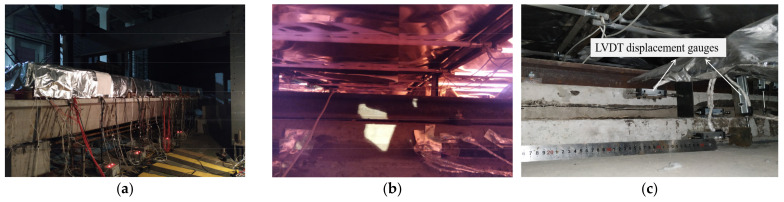
The diagram of the temperature test (**a**) Diagram of thermal loading, (**b**) Diagram of heating process, (**c**) Diagram of LVDT displacement gauges.

LVDT displacement gauges were installed at both sides of the bottom surface of the support bridge, as shown in Figure 5. The diagram of the temperature test is shown in Figure 6.

## 3. Test Results and Analysis

### 3.1. Distribution of Thermal Difference

**Figure 7:**
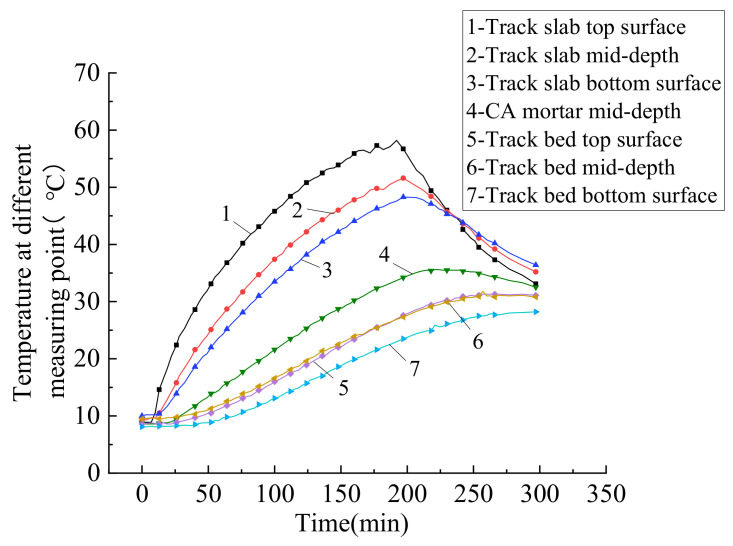
Plots of temperature–time in different layers.

**Figure 8:**
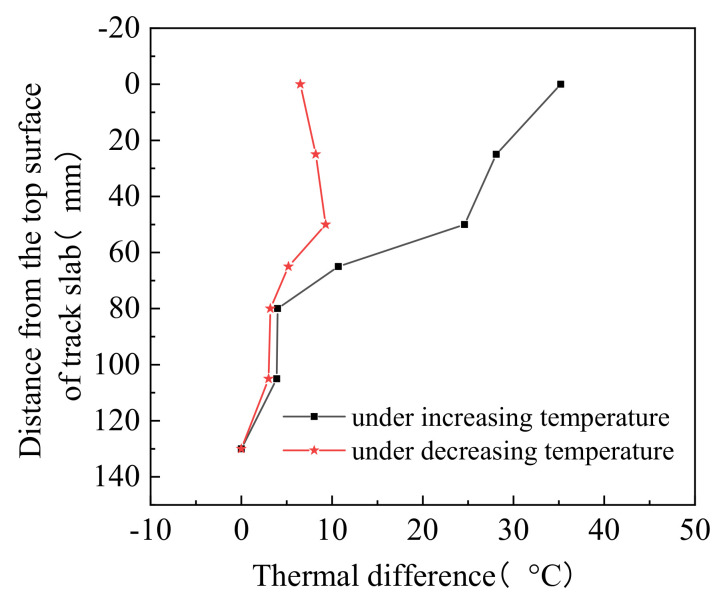
Thermal difference in the vertical direction.

The temperature vs. time diagrams plots shown in Figure 7, where the temperatures of the track layers gradually increase and then gradually decrease, in accordance with the heating and cooling of the top surface. Furthermore, the greater the distance from the top surface of the track slab, the more delayed the occurrence of the peak temperature. According to the change of the track slab top surface temperature with time, the loading process can be divided into two stages: the temperature rising and dropping. In both the heating and cooling phases, the thermal gradients inside the track slab are shown to be higher than those in the other track layers. It can also be seen from Figure 8 that the temperature exhibits a non-linear positive thermal difference distribution from the top of the track slab to the bottom surface of the track bed. Due to the time dependence of temperature transfer inside the track structure, under decreasing temperature, the temperature still exhibited a nonlinear positive thermal difference distribution.

### 3.2. Strain Analysis

Strain in the Layers of Track Structure

**Figure 9:**
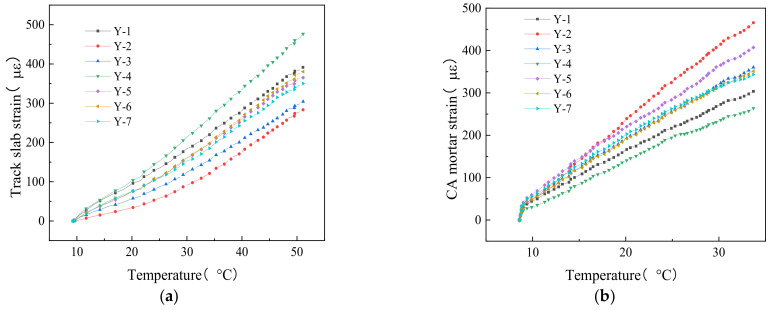

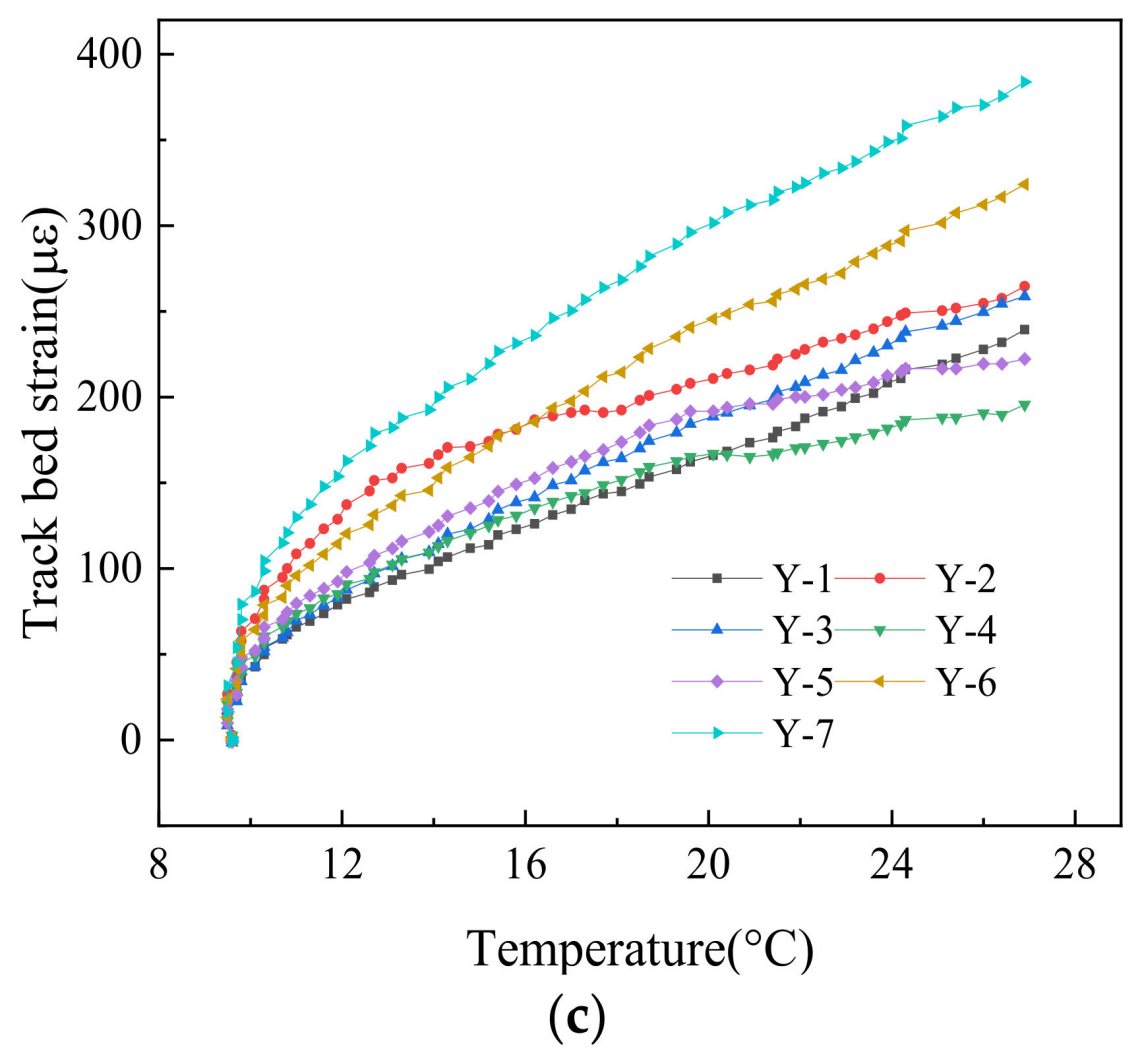
Diagrams of the strain vs. temperature in the track layers: (**a**) track slab, (**b**) CA mortar, and (**c**) track bed.

Figure 9 shows the distribution of strain with temperature in each of the track layers. It can be seen from Figure 9a that the strain in track slab increased nonlinearly under increasing temperature, and the higher the temperature, the greater the tensile strain in the track slab. The gradient of the track slab strain was low at 25 °C. When the temperature reached the highest value, the strain was the highest at all points. At the same thermal load, the strain at mid-span was the largest, and the strain of the end section was the smallest.

Figure 9b shows the plot of the CA mortar layer strain with temperature at different sections. At 10 °C, the CA mortar strain increased rapidly. When the increasing temperature was higher than 10 °C, the CA mortar strain increased nearly linearly to reach maximum. At the same thermal load, the mid-span CA mortar strain was the smallest, and the CA mortar strains at the end sections were the largest.

Figure 9c shows that the track bed strain changed nonlinearly with temperature. During the early period of the heating process, the strain of the track bed increased rapidly, before reaching its maximum with a slow increase rate in the latter period of the heating process.

The strain varied significantly with temperature in all track layers at the monitoring points Y-1 and Y-2. In the track slab, the strain measured at Y-1 was larger than that at Y-2, and the difference of strain was about 100 με between the two points. In the CA mortar and the track bed, the strain measured at Y-2 was larger than that at Y-1, and the difference between the two points was nearly 150 με and 230 με, respectively. The difference of strain observed between these two monitoring points can be explained by the facts that: (1) one of the points was outside the heating area, and the greater the distance from the track slab, the less the thermal transfer effect; and (2) the shear grooves at the fixed end increased the restraint effect between the track bed and the support bridge, therefore the deformation near the fixed-end sections was limited.

The track slab strain curve presents upward concave distribution trend in the longitudinal direction, and that of the CA mortar was the most obvious at the fixed end section. The diagram of the strain in the track slab exhibited upward concavity, similar to the CA mortar strain distribution. The above analysis indicates that relative deformation induced by the thermal load can easily cause gaps between the track slab and the CA mortar layer. In fact, the track structure is exposed to the natural environment, and the cyclic effect of day-by-day thermal load could intensify the relative deformation between track slab and CA mortar. Gaps were developed and underwent three stages: initiation, development, and stability [23]. Under the long-term thermal load, the integrity of the structure will be affected, and the passengers’ comfort and stability will be reduced accordingly.

Overall, under increasing temperature, the strain of track layers showed a nonlinear growth trend. The higher the temperature, the larger the tensile strain, and the tensile strain was the highest in the track slab.

## 4. Displacement Analysis

### 4.1. Relative Displacements between Track Layers

The influence of temperature on the track structure included expansion deformation due to the overall temperature rising and falling, and flexural deformation due to thermal gradient load [11]. Under the irradiation of a far-infrared heating lamp, the upper surface temperature of the ballastless track structure was higher than that of the lower surface, leading to thermal difference or a thermal gradient in the vertical direction. In this paper, the fixed end close to the groove was assumed to be “section 0”, and relative longitudinal displacements were assumed to be positive when their direction was from Section 0 towards the mid-span section. An upward relative displacement was considered positive in the vertical direction.

#### 4.1.1. Relative Longitudinal Displacements between Layers

(1) Displacements between track slab and track bed in the longitudinal direction

**Figure 10:**
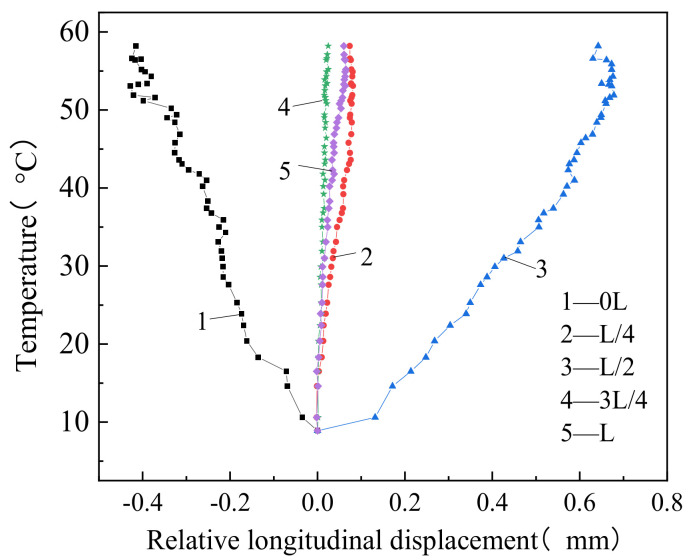
Relative longitudinal displacement between the track slab and track bed.

Figure 10 clearly shows that the longitudinal displacement between the track slab and the track bed increased nonlinearly, because of the heating. When the temperature was the highest, the displacement between the two layers was 0.398 mm toward the mid-span section (negative direction). The relative displacement values between track slab and track bed were 0.075 mm, 0.658 mm, 0.019 mm, and 0.054 mm (positive direction) at the L/4 section, mid-span section, 3L/4 section, and L, respectively. Under increasing temperature, a large displacement was observed between the track slab and the track bed near the fixed end section and at the mid-span section.

Under increasing temperature, the displacement between track slab and track bed measured at the L/4 section was in the opposite direction from that measured at the 0L section; therefore, the zero-displacement point was close to the right side of the L/8 section, where a gap is more likely to initiate [23].

(2) Displacements between the CA mortar and track bed in the longitudinal direction.

**Figure 11:**
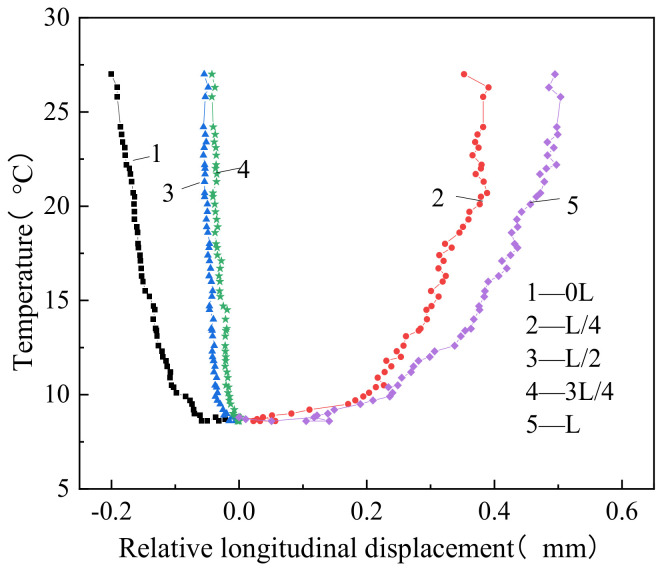
Relative longitudinal displacement between CA mortar and track bed.

From Figure 11, it can be seen that the relative longitudinal displacement between the CA mortar and the track bed grew nonlinearly under increasing temperature. When the temperature reached its highest value, the relative longitudinal displacements between the CA mortar and the track bed were −0.191 mm, 0.382 mm, −0.053 mm, −0.043 mm, and 0.504 mm at the 0L, L/4, L/2, 3L/4and L sections, respectively. The relative longitudinal displacement between these two layers was positive at the L/4 and L sections, and negative at the other sections.

The zero-displacement point between the CA mortar and the track bed occurred at the L/4 and 7L/8 sections, which meant that the CA mortar layer produced two displacement peaks in the longitudinal direction of the beam. Considering that the CA mortar was constrained by the track slab and track bed, combined with the aforementioned displacement mode of the track slab and the stress state of the CA mortar, the upper-arch displacement at the 7L/8 section was not even. In addition, the interlayer bond performance between the CA mortar, the track slab, and the track bed was complex due to the material heterogeneity, which made the displacement distribution between the CA mortar and the track bed non-uniform in the longitudinal direction.

(3) Displacements between the track bed and support beam in the longitudinal direction

**Figure 12:**
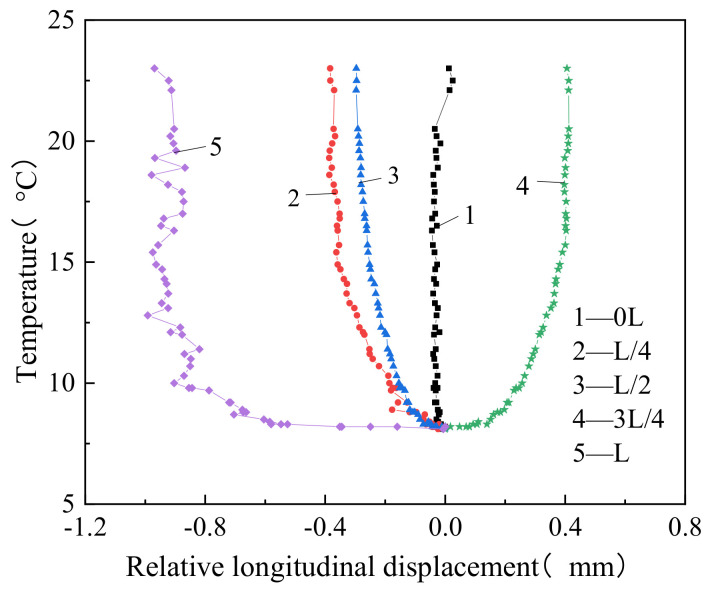
Relative longitudinal displacement between the track bed and support beam.

Figure 12 shows that the relative longitudinal displacement between the track bed and the support beam changed nonlinearly with temperature increase. With the temperature increase, this relative longitudinal displacement increased gradually at all the different sections, except for the sections at both ends, where the relative displacement remained nearly 0. When the temperature reached the highest value, the relative longitudinal displacement values between the track bed and support beam were 0.383 mm (negative), 0.296 mm (negative), and 0.47 mm (positive) at the L/4, L/2, and 3L/4 sections, respectively.

The relative longitudinal displacement between the track bed and the support beam measured at L/4 was of an opposite sign from that measured at the L/2 section; thus, the zero-slip point of the track bed relative to the support beam was near the 5L/8 section, and local extrusion of the track bed caused by the temperature effect made the track bed arch relative to the support beam. According to the above analysis, it can be seen that the restraint effect of the groove on the track bed near the fixed end support was greater than that of the track slab, shifting the zero-slip point of the track bed relative to the support beam to the right.

#### 4.1.2. Relative Vertical Displacement between Layers

(1) Relative vertical displacement between the track slab and the track bed

The relative vertical displacement between the track slab and the track bed increased and reached a peak at the L/4 section, as shown in Table 3a. The relative vertical displacement between track slab and track bed followed the increase in temperature, to increase from the end sections to the mid-span section. The track slab had an upward deflection at the L/4 section. With a temperature increment of 10 °C, the relative vertical displacement at the L/4 section was 0.41 mm, 0.43 mm, 0.55 mm, 0.60 mm and 0.70 mm, respectively, i.e., the vertical relative displacement between the track slab and the track bed was 4.87%, 27.9%, 9.09% and 16.7% of the previous temperature rise condition, respectively, on the L/4 section for every 10 °C temperature rise.

In the vertical direction, a positive thermal gradient occurred in the track structure. This moved the track slab upward relative to the track bed with a peak value at the L/4 section.

(2) Relative vertical displacement between track bed and support beam

The relative vertical displacement between the track bed and the support beam increased with temperature, as shown in Table 3b. The vertical displacement between the track bed and the support beam did not exceed 0.5 mm at the end sections. With a temperature increment of 10 °C, the relative vertical displacement on the 3L/4 section was 1.052 mm, 1.291 mm, 1.580 mm, 1.750 mm and 1.900 mm, respectively, i.e., the vertical relative displacement increment between the track bed and the support beam was 22.7%, 22.4%, 10.8% and 8.6% compared with the previous temperature rise condition for every 10 °C temperature rise.

Under the action of positive thermal gradient, the relative vertical displacement between the track layers increased continuously. The relative vertical displacement was nearly 0 at the end sections, while a significant increase was observed at the mid-span section. Therefore, in the design of ballastless track–HSR bridge structural systems, the vertical contact between layers should be considered to ensure that relative vertical displacement between the track layers stays within the design requirements under high thermal load.

To sum up, under increasing temperature, expansion occurs in the track layers. The zero-displacement point between the track slab and the track bed, and the track bed and the support beam were located at the L/8 section and 3L/8 section, respectively. It is clear that thermal load on the track structure resulted in large displacement between the track layers, which resulted in interlayer gap initiation. In practice, the track structure was cyclically and continuously exposed to day–light thermal loads, which allowed interlayer gaps to initiate, propagate, and stabilize.

### 4.2. Overall Displacement of the Support Beam

**Figure 13:**
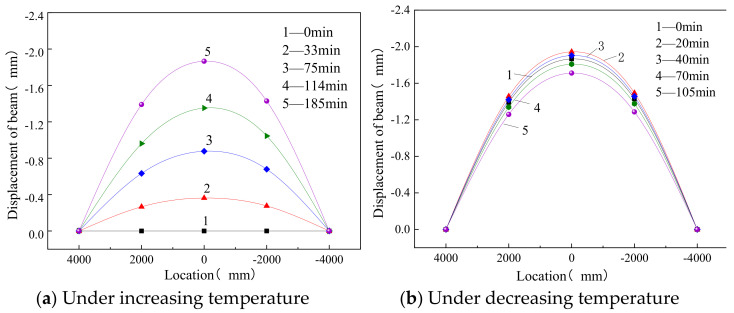
Displacement of the beam under different conditions.

The displacements of the supporting beams in thermal conditions are plotted in Figure 13, where at increasing temperature, the deformation measured at the bottom of beam mid-span was greater than that measured at the beam end supports. When the top surface of the track slab was heated to the maximum temperature of 58.2 °C, the displacement of the support beam reached its maximum value. Additionally, the value in mid-span was 1.942 mm.

The thermally induced camber of the track structure tended to force the supporting beam upwards, which, in turn, tended to restrain any upward displacement of the track structure.

It can be seen that under increasing temperature, the beam gradually arched, and the higher the temperature, the greater the displacement of the support beam. Under decreasing temperature, the camber of the beam decreased slowly. Considering that there was no bond between the support beam and the track structure, the deformation of the beam was only caused by thermal expansion and contraction when it was subjected to the thermal load. In addition, the maximum camber displacement of the track structure was greater than that of the support beam. The restraint effect and thermal gradient effect were the mean reasons for this result.

## 5. Stress Analysis

### 5.1. Formulation and Validation of the Numerical Model

#### 5.1.1. Model Formulation

A three-span ballastless track structure was modeled by a finite element analysis software, implemented with the constitutive materials models proposed in [27]. The geometry of the track structure in the FE model strictly adhered to that of the test specimen. The most relevant parameters are reported in Table 4. The contact among the different layers was modeled by means of interface cohesive elements, while nonlinear springs were introduced to describe the contact between the track structure and the underlying supporting structure.

**Figure 14:**
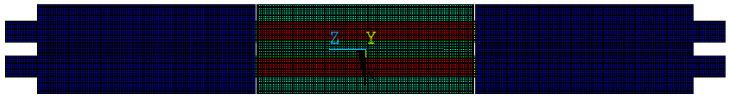
FE discretization of the track–bridge structure.

**Figure 15:**
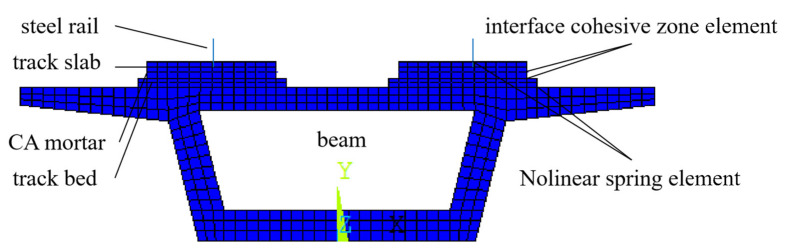
FE discretization of the cross section of the track–bridge structure.

Figure 14 shows the top view of the model, and Figure 15 shows the cross section of the structure.

#### 5.1.2. Model Validation

To keep the numerical model as close as possible to the test model, the thermal load was introduced in full agreement with the procedure adopted in the test, as shown in Table 5.

**Figure 16:**
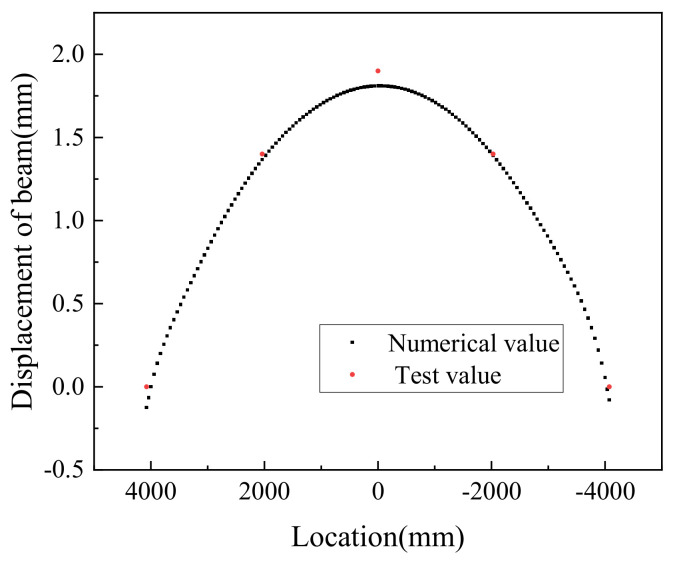
Numerical and experimental values of beam displacements.

Figure 16 shows the numerical and tested values of beam displacement at 50 °C of heating. The plot shows that when the temperature was raised to 50 °C, the numerical value of the upper arch of the beam was in good agreement with the test value, and the deflection test value and the calculated value of the beam at the middle span were 1.942 mm and 1.81 mm, respectively. The error was 7.3%, which is less than 10%, meaning that the main parameters of the finite element model in this paper are reasonable and the finite element calculation results are suitable.

### 5.2. Comments on the Results

#### 5.2.1. Positive Thermal Gradients

To study the influence of thermal load on the ballastless track–bridge structural system, the top surface of the track slab was taken as a starting point in the loading process, and Table 6 shows the loading conditions in the numerical model.

**Figure 17:**
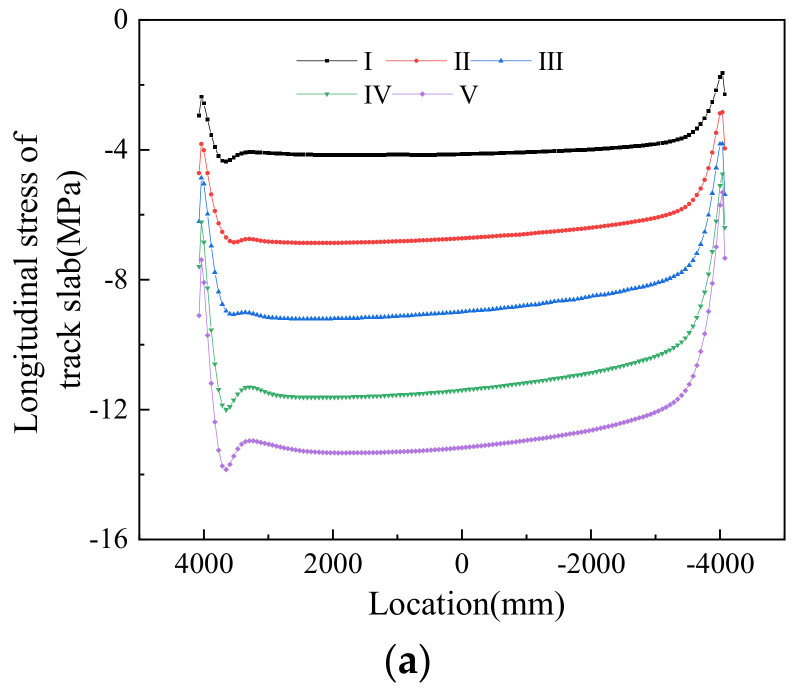

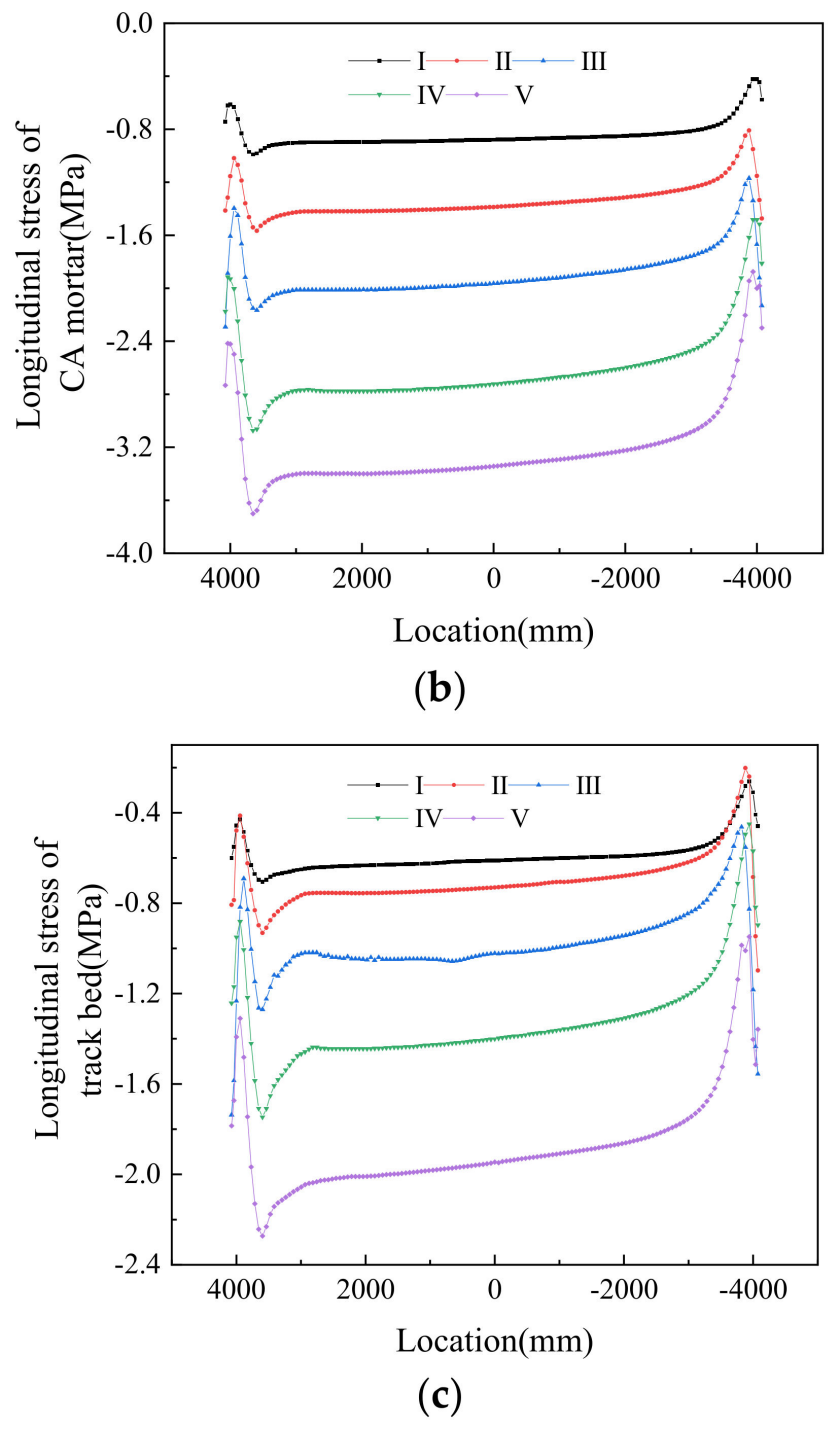
Stress profiles of track structure in the longitudinal direction under positive thermal gradients: (**a**) track slab, (**b**) CA mortar, and (**c**) track bed.

**Figure 18:**
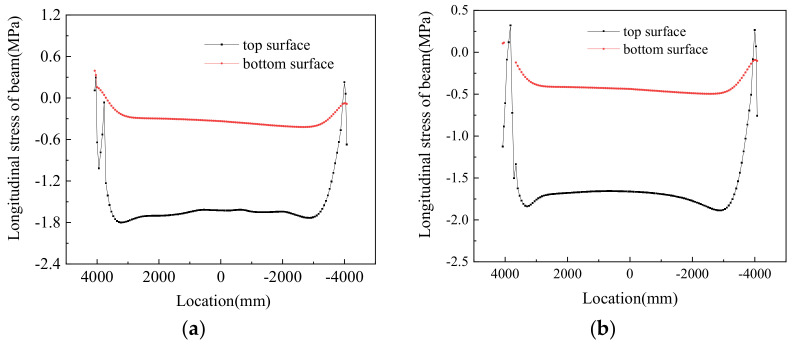

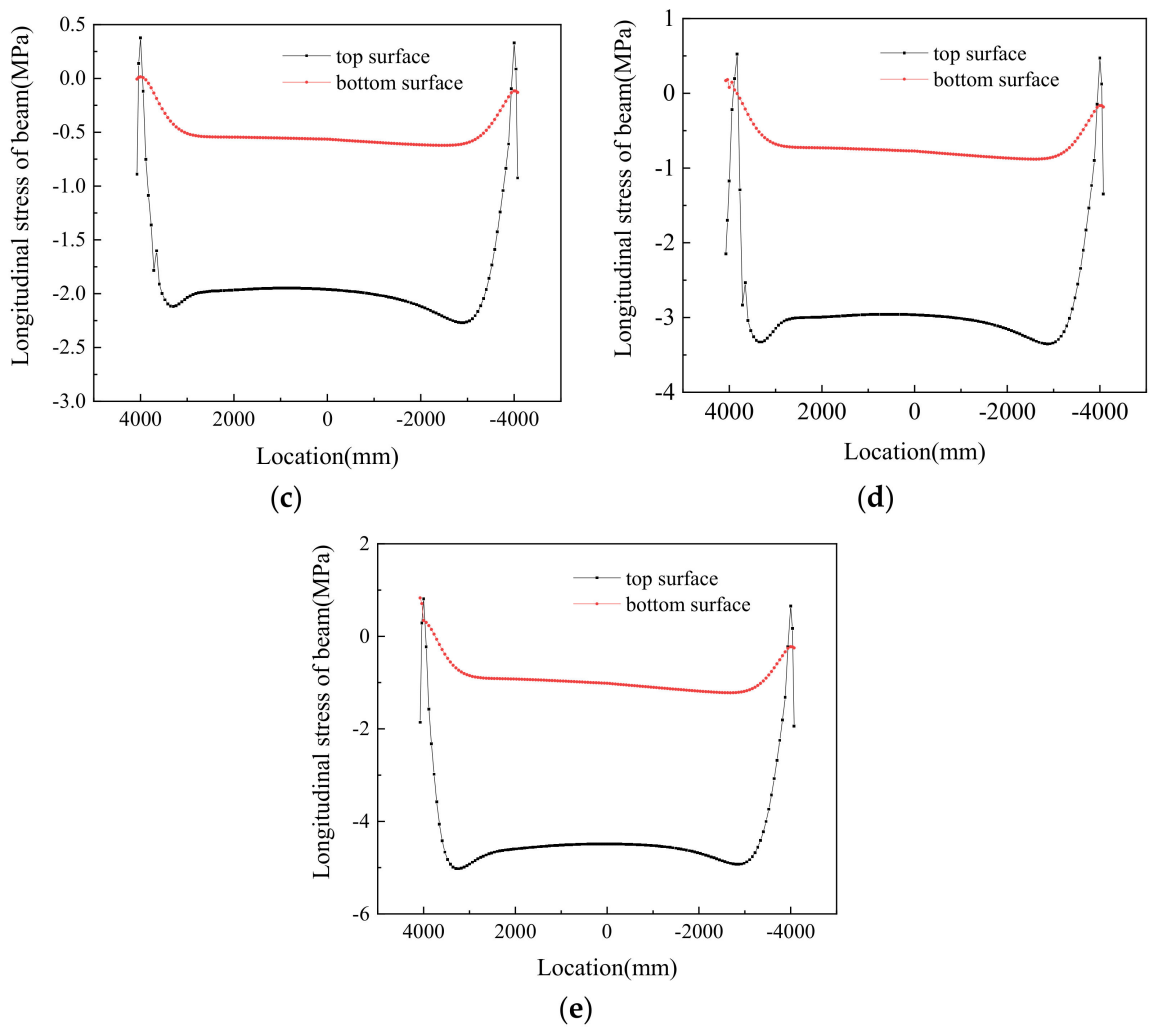
Numerical values of the stress in the supporting beams under positive thermal gradients: (**a**) I, (**b**) II, (**c**) III, (**d**) IV, and (**e**) V.

Figure 17 shows profiles of track structure stress in the longitudinal direction under different working conditions. It can be seen from the plot that under positive thermal gradients, the larger the positive thermal gradient, the greater the stress of the track structure. Figure 18 shows the stress profiles of the beam in the longitudinal direction under different working conditions. It can be seen from the plot that the stress of the top surface on the beam is much larger than the longitudinal stress of the bottom surface of the beam.

Table 7 shows the maximum stress of the track–bridge structure in the longitudinal direction under positive thermal gradients. According to the data in the table, under the same thermal gradient, the maximum stress occurred on the track slab, and the minimum stress appeared on the bottom surface of the beam.

The maximum stress values shown in Table 7 were on the section where the grooves were located. With the increase in positive thermal gradient, the stress at the grooves’ sections also increased in the different structural layers.

#### 5.2.2. Negative Thermal Gradients

**Figure 19:**
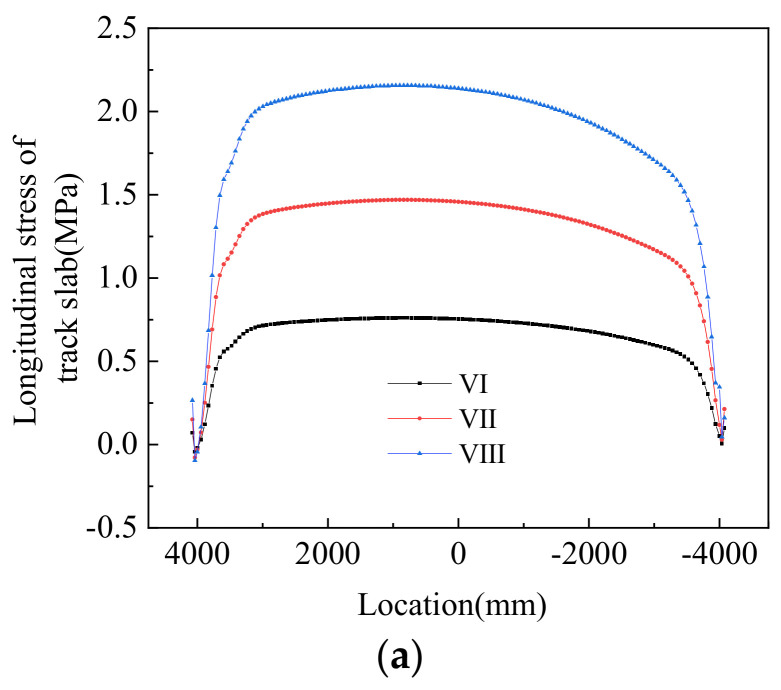

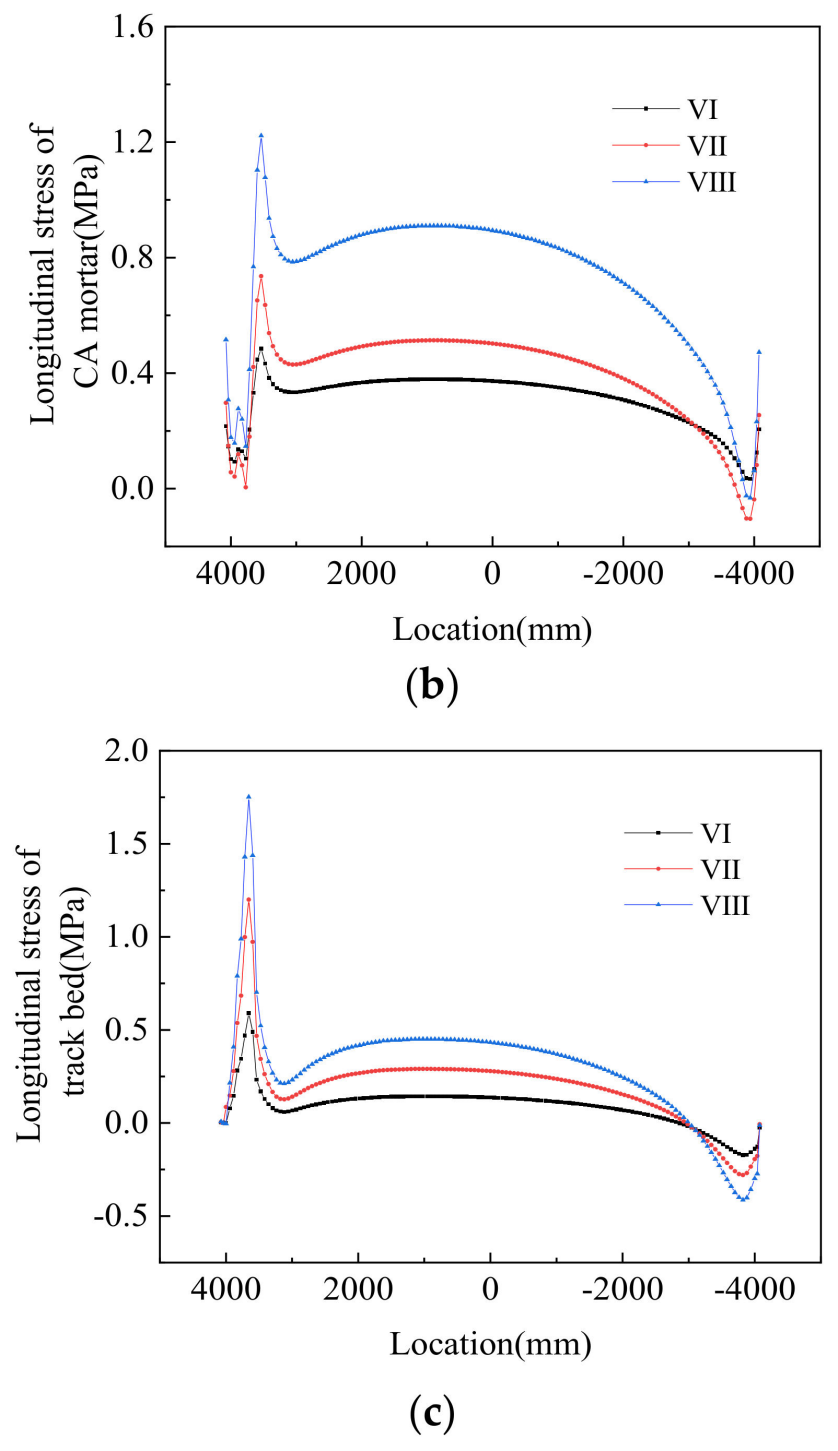
Stress distribution of the track structure under negative thermal gradients: (**a**) track slab, (**b**) CA mortar, and (**c**) track bed.

**Figure 20:**
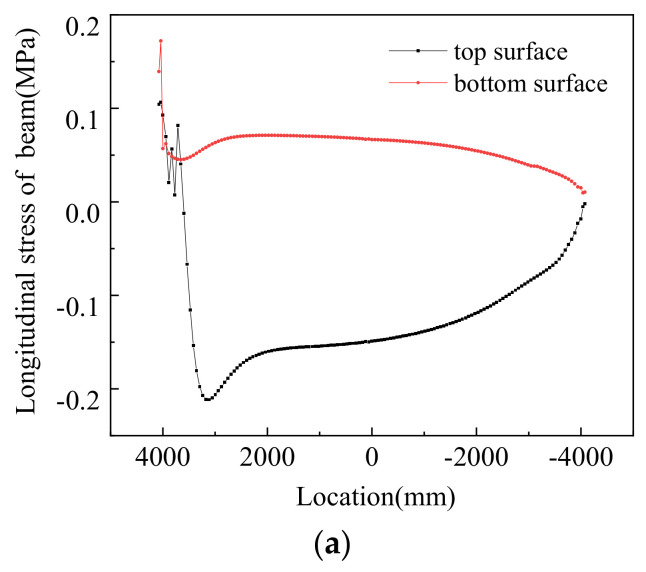

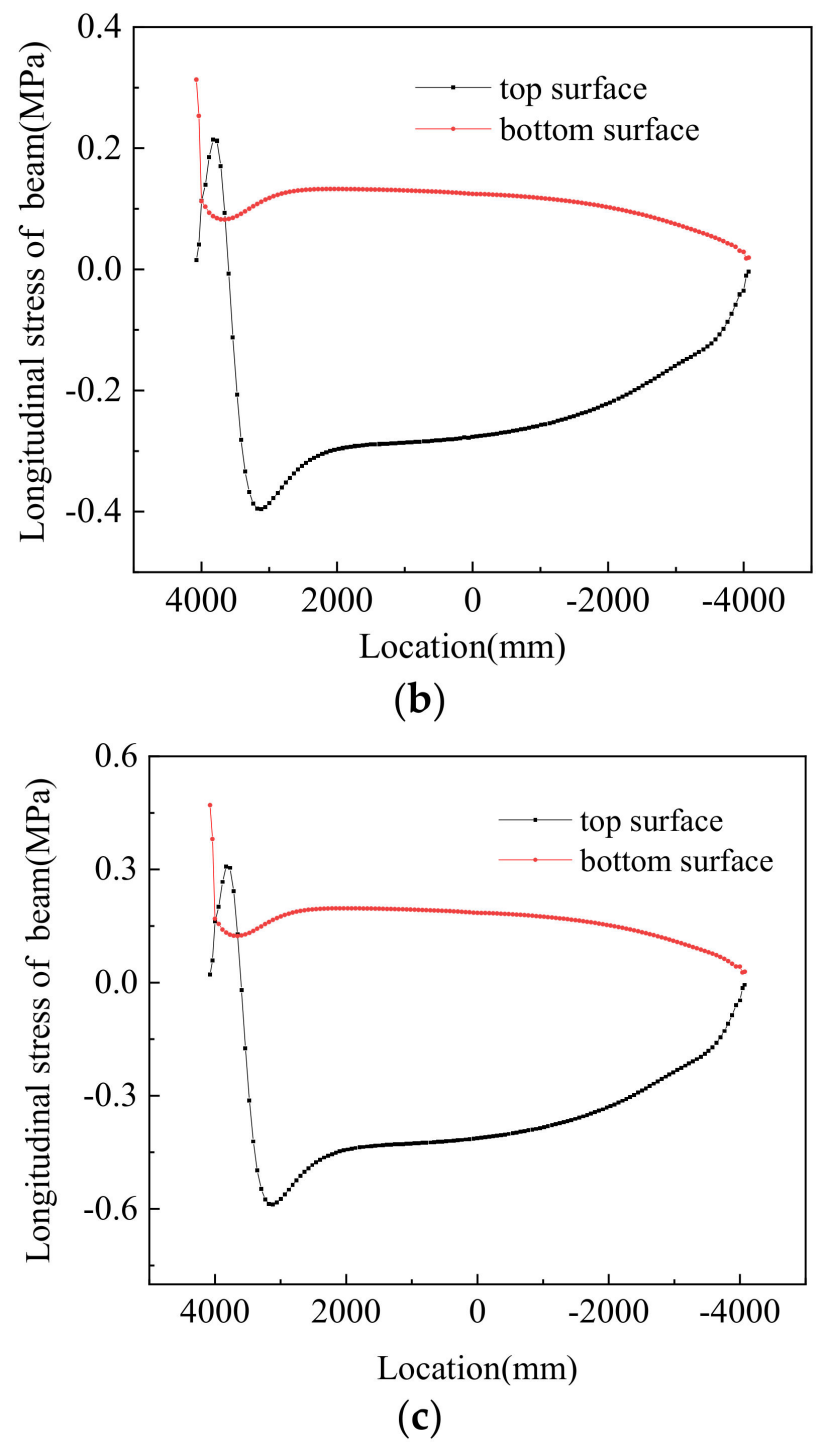
Numerical results concerning the stresses in the bridge beam under negative thermal gradients: (**a**) VI, (**b**) VII, and (**c**) VIII.

Figure 19 shows the stress profiles in the track structures under negative thermal gradients. It can be seen from the plot that under negative thermal gradients, the system was mainly tensile, and the maximum tensile stress of 2.156 MPa appeared on the track slab. This is lower than the standard value of compressive strength of C50 concrete. Figure 20 shows the longitudinal stress distribution on the beam under different loading conditions. It can be seen from the diagram that under negative thermal gradients, the top surface of the beam was mainly under compression, and the bottom surface of the beam was mainly pulled. Table 8 shows the longitudinal stress under different working conditions of the track–bridge structure under negative thermal gradients.

To sum up, decreasing temperatures induced higher tensile stresses in the track structure, as well as larger displacements. It is, therefore, imperative to have a clear picture of the stress and displacement fields in extreme thermal conditions during the design phase of ballastless track structures for high-speed railway lines.

## 6. Conclusions

1. The experimental and numerical study carried out in this project regarding thermal loads in a ballastless track system for the railway bridges of high-speed railway lines makes it possible to draw the following conclusions: the strains in the track layers increase nonlinearly with temperature; the higher the temperature, the larger the tensile strain, and the maximum tensile strain in the track slab is higher than the other layers; the relative displacements between track slab and cement-emulsified asphalt (CA) mortar is the driving force behind heat-induced gap initiation;
2. Under increasing temperature, the relative displacement of the different layers of track structure in the longitudinal direction is nonlinear. Due to the shear grooves, the relative zero-displacement point between the track slab and track bed, between the CA mortar and track bed, and between the track bed and the supporting beam changes continuously from the fixed end section to sliding end section in the longitudinal direction. The plot of vertical relative displacement in track–bridge structures with temperature is consistent with that of longitudinal relative displacement;
3. The overall displacement of the beam increases gradually under increasing temperature. The overall displacement of the beam decreases slowly under decreasing temperature. The profiles of the vertical displacement in the supporting beams clearly indicate a camber due to the positive thermal gradients;
4. Under positive thermal gradients, the track structure is under compression. The larger the positive thermal gradient, the greater the compressive stress in the track structure. Under negative thermal gradients, the track structure is pulled downwards by the supporting structure, and negative thermal gradients have a great influence on the internal force of the track structure, which makes the track structure layer produce larger tensile stress.

## Figures and Tables

**Table 1:** Similarity ratio of physical quantity in test structure and prototype structure.

Physical Quantity	Dimension	Test Structure	Prototype Structure	Similarity Ratio
Physical dimension	L	l/4	1	1/4
Modulus of elasticity	M/(L*T^2^)	E	E	1
Coefficient of linear expansion	1/θ	α	α	1
Temperature	θ	T	T	1
Thermal gradient	θ/L	∇T	4∇T	1/4

**Table 2:** Similarity ratios for the physical quantity appearing in the test.

Physical Quantity	Dimension	Test Structure	Prototype Structure	Similarity Ratio
Stress	M/(L*T^2^)	σ	σ	1
Strain	1	ε	ε	1
Displacement	L	δ/4	δ/4	1/4

**Table 3:** Relative vertical displacement of the structure in the longitudinal direction.

(a) Between the track slab and track bed (mm).				
0L	L/4	L/2	3L/4	L
0.030	0.410	0.370	0.420	0.180
0.040	0.430	0.410	0.500	0.420
0.300	0.550	0.180	0.200	0.250
0.470	0.600	0.550	0.300	0.310
0.400	0.700	0.500	0.420	0.400
(**b**) Between the track bed and support beam (mm).				
**0L**	**L/4**	**L/2**	**3L/4**	**L**
0.010	0.065	0.077	1.052	0.049
0.086	0.163	0.366	1.291	0.066
0.012	0.252	0.670	1.580	0.273
0.031	0.316	1.031	1.750	0.352
0.247	0.348	0.687	1.900	0.418

**Table 4:** Parameters of the FE model.

Structure	Unit Type	Modulus of Elasticity (Pa)	Poisson Ratio	Density (kg/m^3^)	Linear Expansivity (1/°C)
Steel rail	BEAM189	2.10 × 10^5^	0.3	7800	1.20 × 10^−5^
Track slab (C55)	SOLID65	3.60 × 10^4^	0.2	2600	1.00 × 10^−5^
CA mortar	SOLID65	1.17 × 10^4^	0.2	1800	1.00 × 10^−5^
Track bed (C30)	SOLID65	3.20 × 10^4^	0.2	2600	1.00 × 10^−5^
Beam (C50)	SOLID65	3.55 × 10^4^	0.2	2600	1.00 × 10^−5^
Bridge abutment	SOLID65	2.63 × 10^4^	0.2	2600	1.00 × 10^−5^
Tendon	LINK8	1.95 × 10^5^	0.3	7800	1.20 × 10^−5^

**Table 5:** Thermal load.

Structure	Temperature Increased by 50 °C
Top of track slab	58.2 °C
Bottom of track bed	37.6 °C
Bottom of CA mortar	27 °C
Bottom of track bed (Top surface of beam)	23 °C
200 mm from the top of beam	0 °C

**Table 6:** Loading conditions.

Load Condition	І	Ⅱ	Ⅲ	Ⅳ
Thermal gradient (°C/m)	20 °C/m	40 °C/m	60 °C/m	80 °C/m
**Load Condition**	**Ⅴ**	**Ⅵ**	**Ⅶ**	**Ⅷ**
Thermal gradient (°C/m)	100 °C/m	−10 °C/m	−20 °C/m	−30 °C/m

**Table 7:** Compressive stress under different conditions in the track–bridge structure (MPa).

Load		Track Slab	CA Mortar	Track Bed	Top Surface of Beam	Bottom of Beam
І	maximum	−4.366	−0.990	−0.706	−1.780	−0.420
	minimum	−1.634	−0.422	−0.262	−0.084	−0.089
Ⅱ	maximum	−6.699	−1.540	−0.931	−1.888	−0.800
	minimum	−2.844	−0.951	−0.240	−0.084	−0.105
Ⅲ	maximum	−8.968	−2.152	−1.270	−2.270	−0.622
	minimum	−3.819	−1.339	−0.825	−0.096	−0.006
Ⅳ	maximum	−12.008	−3.074	−1.746	−3.310	−0.881
	minimum	−4.742	−1.482	−0.450	−0.148	−0.067
Ⅴ	maximum	−13.845	−3.701	−2.273	−5.025	−1.220
	minimum	−5.309	−1.875	−0.949	−0.228	−0.064

**Table 8:** Longitudinal stress under different conditions of the track–bridge structure (MPa).

Load		Track Slab	CA Mortar	Track Bed	Top Surface of Beam	Bottom of Beam
Ⅵ	maximum	0.760	0.484	0.590	0.106	0.172
	minimum	−0.044	0.034	−0.172	−0.212	0.010
Ⅶ	maximum	1.469	0.736	1.120	0.214	0.313
	minimum	−0.078	−0.104	−0.280	−0.396	0.018
Ⅷ	maximum	2.156	1.222	1.752	0.308	0.470
	minimum	−0.096	−0.031	−0.413	−0.588	0.027

## Data Availability

Data sharing is not applicable.

## Nomenclature

σ	Stress
$\sigma_{x}^{ꞌ}$	Horizontal axial stress in the full-scale model
$\varepsilon_{x}$	Horizontal axial strain in the scale model
*E*	Elastic modulus
*L*	Span of the specimen
ΔT	Thermal difference in the vertical direction
θ	Thermal
*M*	Mass
$\sigma_{x},\sigma_{y},\sigma_{z}$	Stress in different directions in the scale model
ε	Strain
T	Time
$\varepsilon_{x}^{ꞌ}$	Horizontal axial strain in the full-scale model
μ	Poisson’s ratio
α	Linear expansion coefficient
∇T	Thermal gradient
$c_{x}$	Similarity constant
δ	Displacement
